# Chemistry of adaptive polymer-based protective coatings for harsh environments: chemical aging, anticorrosion, antifouling, and self-healing design

**DOI:** 10.3389/fchem.2026.1910772

**Published:** 2026-10-20

**Authors:** Stefano Bellucci

**Affiliations:** 1 Qi S.r.l., Pomezia, Italy; 2 Universidad Ecotec, Samborondón, Ecuador; 3 Nano Research Laboratory, Excellent Center, Baku State University, Baku, Azerbaijan

**Keywords:** adaptive coatings, anticorrosion, antifouling, chemical aging, electrochemical impedance spectroscopy, polymer chemistry, responsive polymer networks, self-healing

## Abstract

Polymer-based protective coatings are moving from passive barriers toward adaptive chemical interfaces that regulate transport, adhesion, inhibitor release, fouling resistance and damage recovery under harsh service conditions. This review examines organic and polymer-rich coatings designed for corrosive, marine, humid, acidic, alkaline and biologically active environments from a polymer and surface chemistry perspective. Epoxy, polyurethane, acrylic, siloxane, catechol-containing, zwitterionic, hydrogel-like and bio-derived coatings are discussed as platforms in which network architecture, crosslink density, wet adhesion, free volume, interfacial polarity and surface hydration determine long-term performance. Selected graphitic-polymer studies are used as metrology and corrosive-aging precedents, while the main evidence base is the international literature on active anticorrosion, nanocontainer release, dynamic covalent and supramolecular self-healing, antifouling surfaces and accelerated validation. The unique contribution is a failure-sequence and evidence-hierarchy framework linking barrier degradation, inhibitor release, self-healing and antifouling into a single validation logic. The review argues that adaptive coatings should not be evaluated only by initial impedance, water contact angle, corrosion current or scratch closure. Their value depends on whether protective function is retained after coupled aging, mechanical damage, electrolyte exposure, fouling pressure and repeated wet-dry or UV-salt-humidity cycling.

## Graphical Abstract

**Figure:**
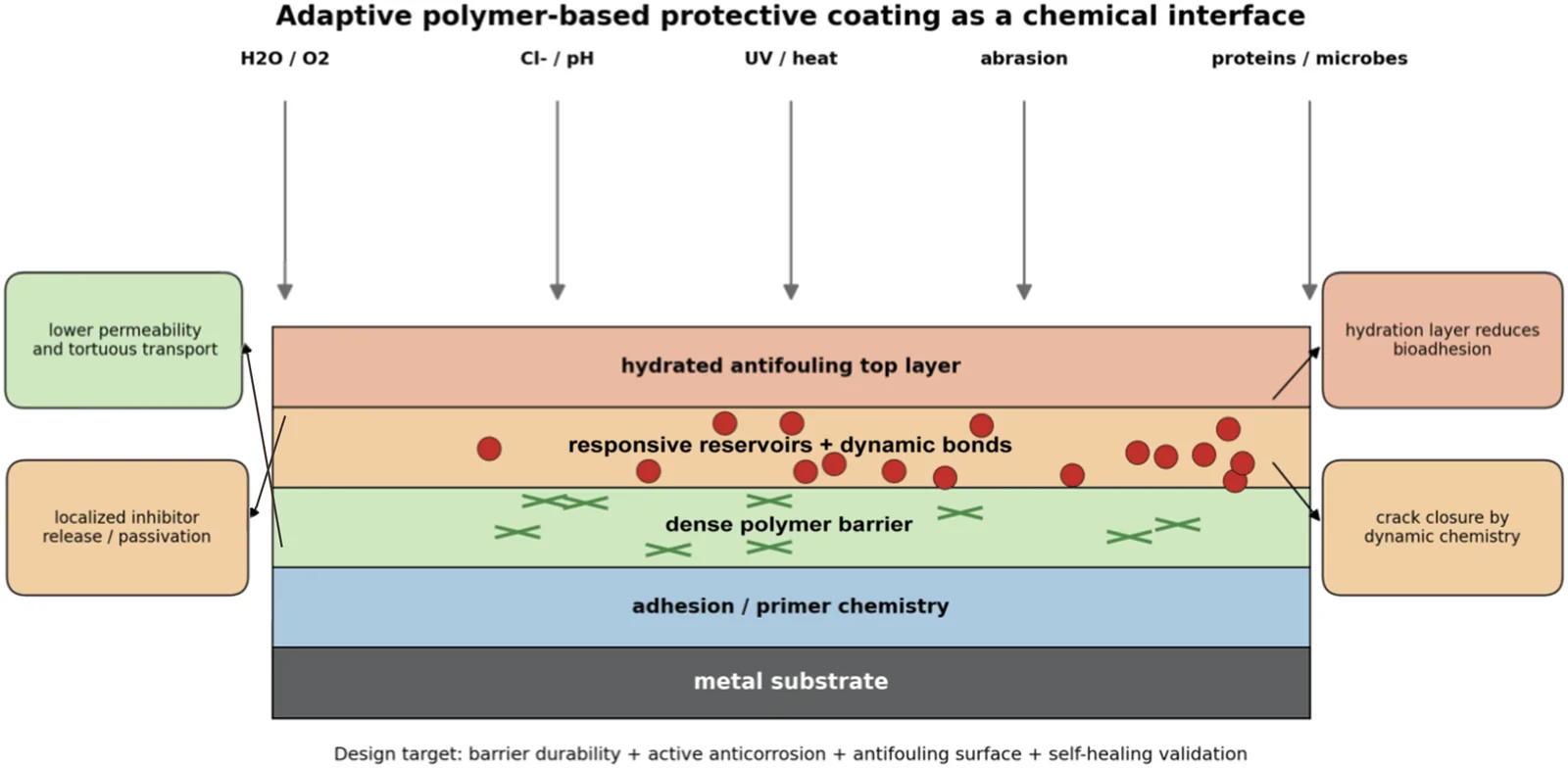
Adaptive protective coatings are treated as stratified chemical interfaces that combine substrate adhesion, dense polymer barrier transport, responsive inhibitor or self-healing domains, and hydrated antifouling surface chemistry. The scheme is original and summarizes the design logic discussed in this review.

## 1 Introduction: from passive coatings to adaptive chemical interfaces

Corrosion, biofouling and chemical aging are often treated as separate coating problems, but in service they are coupled through water uptake, ion transport, interfacial contamination, mechanical damage and progressive loss of wet adhesion. A coating exposed to chloride solution, cyclic humidity, ultraviolet radiation, temperature variation, acidic or alkaline environments and biological conditioning does not fail through one mechanism at a time. It fails through a sequence in which polymer swelling accelerates ion diffusion, diffusion weakens the coating/metal interface, interfacial defects concentrate electrochemical activity, and damaged regions become more susceptible to fouling or further chemical attack.

This review defines an adaptive polymer-based protective coating as an organic or polymer-rich surface layer that does more than slow permeation at time zero. An adaptive coating changes its local function when damage or environmental triggers appear. It may release an inhibitor at a corrosion site, restore barrier continuity after a scratch, reorganize hydrogen-bonding or metal-coordination crosslinks, replenish a low-adhesion surface, resist protein or microbial conditioning layers, or provide diagnostic information through impedance or color change. These functions are chemical responses, not merely property labels. The layer-level design logic for this adaptive coating concept is summarized schematically in Graphical abstract.

The discussion follows a chemistry-centered structure: molecular design of polymer networks, interfacial adhesion, barrier transport, responsive release, self-healing chemistry, antifouling surface hydration, electrochemical diagnostics and aging validation. Selected conductive or inorganic additives are considered only where they affect coating-specific questions such as barrier tortuosity, inhibitor storage, wet adhesion, surface hydration, damage recovery or metrology. This concise boundary keeps the review centered on polymer and surface chemistry rather than on device-level metrics.

Several graphitic-polymer studies provide useful background for coating durability and metrology. Work on CNT composites for aerospace applications highlighted the translation value of polymer composites under structural and service constraints (Bellucci et al., 2007). Epoxy/graphene nanoplatelet and exfoliated graphite studies showed the importance of synthesis, dispersion and electrical connectivity in polymer matrices (Dabrowska et al., 2014; Plyushch et al., 2016; Ivanov et al., 2017). Thermal and microwave characterization of graphene nanoplatelets illustrated how measurement geometry, anisotropy and contact quality affect reported properties (Potenza et al., 2017; Bellucci et al., 2018; Bellucci et al., 2019). Most directly, the study of graphene epoxy/nanoplatelet thin films aged in H2SO4 and KCl environments provides an explicit example of coating-relevant chemical aging under corrosive media (Bellucci, 2022). These papers are cited as technical precedents for processing, metrology and aging, while the coating argument is built mainly from the wider literature on polymer protection.

The novelty of this review lies in connecting four literature that are often separated: anticorrosion barrier coatings, active inhibitor-release systems, self-healing polymer networks and antifouling polymer surfaces. The thesis is practical: a future coating paper should not claim application readiness from a single test. A convincing adaptive coating must show an integrated sequence from chemistry to architecture, from architecture to transport control, from transport control to damage response, and from damage response to validated durability. Graphical abstract summarizes the proposed adaptive coating architecture, while Figure 1 summarizes the coupled failure pathway and the points where adaptive design can interrupt it.

**FIGURE 1:**
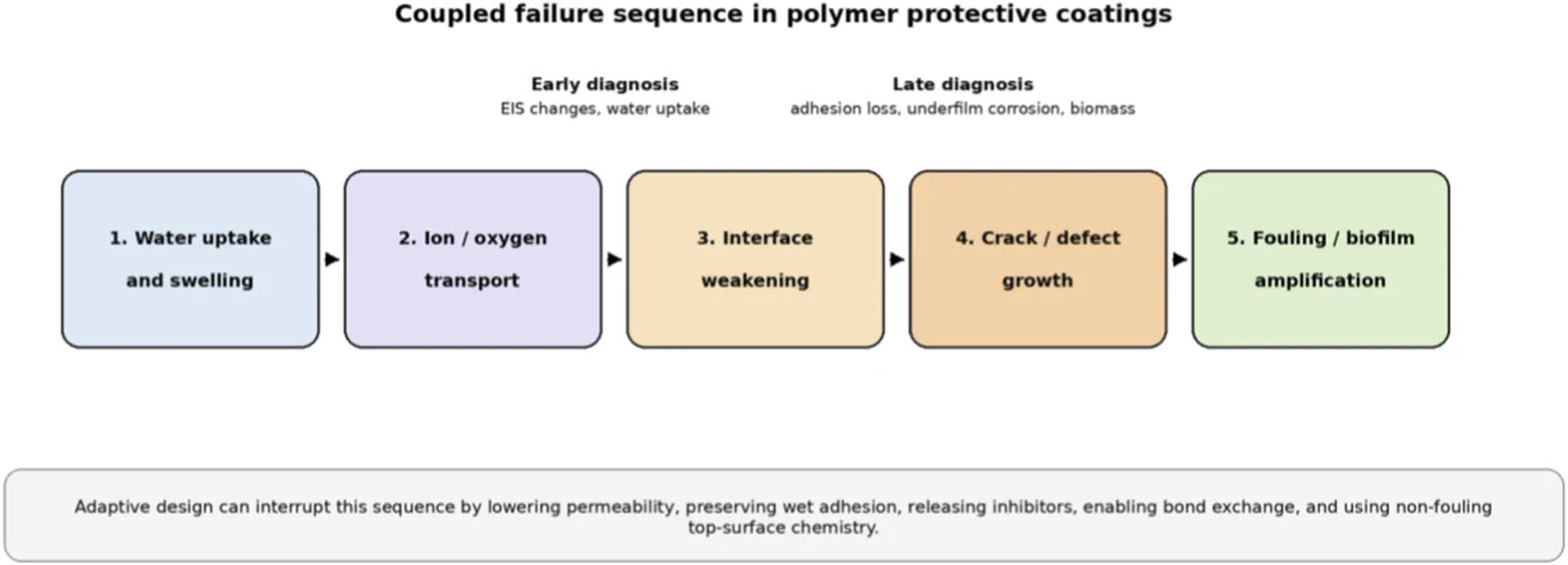
Coupled failure sequence in polymer protective coatings. Water uptake, transport, interfacial weakening, mechanical defect growth and biological amplification should be tested as a linked sequence rather than as independent phenomena.

## 2 Scope and literature selection

The scope covers polymer-based coatings and surface layers designed for aggressive environments. Polymer-based includes epoxy, polyurethane, acrylic, polysiloxane, fluorinated, catechol-containing, zwitterionic, hydrogel-like and bio-derived matrices, plus polymer-rich hybrids in which the polymer network governs adhesion, transport and surface response. The review includes nanofillers and inorganic containers only when they serve coating-specific chemistry, for example by increasing tortuosity, carrying inhibitors, improving wet adhesion, introducing pH response or supporting a recoverable barrier after damage.

To avoid repetition, the scope boundary is stated once here. The review is restricted to polymer-based and polymer-rich coating systems in which formulation chemistry, interfacial adhesion, transport, active release, surface hydration or validation determines protective performance. Additives such as graphene derivatives, oxides, layered hydroxides, clays, metal-organic frameworks or capsules are included only when they perform a coating role rather than serving as a separate materials taxonomy. This scope is consistent with recent assessments of smart protective coatings that integrate framework materials, two-dimensional nanomaterials and carbonaceous structures as functional components within coating architectures (Ghaderi et al., 2024).

The literature base was assembled around formulation chemistry and validation. Priority was given to studies that connect coating composition with dry-film thickness, curing, dispersion, microstructure, inhibitor loading, release behavior, scratch response, impedance recovery, salt or immersion aging, adhesion retention and fouling assays. Reviews are used for field context, while the tables emphasize experimentally grounded coating studies and their limits.

To strengthen the field-wide literature base, additional primary papers are incorporated on inhibitor-loaded microcapsules, dual-pH responsive coatings, micro/nanocontainer release, liquid-infused surfaces, pH-responsive hydrogel microdomains, plastron stability and data-driven aging prediction. Representative examples include linseed-oil and diisocyanate microcapsules (Suryanarayana et al., 2008; Zhang et al., 2024), water-resistant reactive microcapsules (Sun et al., 2016), nanodiamond and layered-double-hydroxide nanocontainers (Rahmani et al., 2020; Xu et al., 2024), dual-pH sensitive inhibitor-loaded coatings (Li et al., 2021), and recent approaches to combined aging prediction and surface-state validation (Chen et al., 2025; Tesler et al., 2024).

## 3 Polymer matrices: chemistry, advantages and failure modes

Epoxy coatings remain the dominant industrial platform for corrosion protection because they combine adhesion, chemical resistance, dimensional stability and compatibility with pigments, inhibitors and reinforcing additives. Their performance is governed by crosslink density, cure conversion, free volume, residual stress, stoichiometry, filler dispersion and substrate pretreatment. High crosslink density can lower permeability but increase brittleness and residual stress. Lower crosslink density can improve toughness but increase water uptake and plasticization. These trade-offs explain why thickness, curing history and environmental exposure must be reported together rather than treated as secondary experimental details (de Paula et al., 2024).

Polyurethane coatings provide elasticity, abrasion resistance and tunable segmental morphology. The soft/hard segment structure offers a chemical basis for dynamic hydrogen bonding, disulfide exchange, urethane exchange and shape-memory-assisted healing. Polyurethanes are therefore attractive for flexible or impact-prone surfaces, but hydrolysis, UV aging and changes in mechanical properties during long immersion can compromise durability. Magnetically responsive, photothermal or dynamically bonded polyurethane systems are recent examples of attempts to combine mechanical compliance with active protection, but the field still needs more realistic aging sequences and larger-area coating demonstrations.

Acrylic, fluorinated and polysiloxane coatings are often selected for weatherability, transparency, low surface energy or fouling release. Fluorinated surfaces can reduce adhesion of organisms and contaminants, but persistence and environmental concerns push the field toward lower-fluorine or fluorine-free alternatives. Polysiloxanes provide low surface energy and high flexibility, while zwitterionic and hydrogel-like coatings work by forming strongly hydrated interfacial layers. These materials are central to antifouling chemistry because they alter the earliest surface-conditioning events rather than simply killing organisms after attachment.

Catechol-containing and polydopamine-inspired coatings connect adhesion, wet surface chemistry and dynamic interactions. Catechol groups can bind metal oxides, coordinate metal ions and participate in reversible networks. The same chemistry appears in self-healing anticorrosion polymers that use catechol-metal coordination as a dynamic crosslinking motif (Song et al., 2023). The challenge is that catechol oxidation, pH sensitivity and uncontrolled metal coordination can alter long-term behavior. The advantage is that these chemistries make adhesion and healing part of the same molecular design problem.

Bio-derived coatings based on lignin, tannic acid, plant polyphenols, cellulose nanofibrils, chitosan derivatives, alginate derivatives, fatty-acid-derived resins or bio-based epoxies are increasingly attractive because protective coatings are under pressure to reduce volatile organic compounds, persistent biocides and hazardous inhibitors. Their success cannot be judged only by renewability. A bio-based coating must still meet the same standards for adhesion, permeability, hydrolytic stability, fouling resistance, release control and end-of-life safety.

A useful way to compare polymer matrices is to ask which molecular motion is allowed after curing. Highly crosslinked epoxies give strong barrier performance but limited segmental mobility; segmented polyurethanes tolerate deformation but may hydrolyze; siloxanes lower adhesion of fouling organisms but often need primers; zwitterionic and hydrogel-like layers maintain hydration but must be mechanically anchored. This molecular-motion view explains why a coating architecture often separates roles into layers: an adhesion-promoting primer, a dense barrier layer, a responsive active layer and a surface layer optimized for wetting or fouling release. Table 1 compares these coating families by useful chemistry, main vulnerability and adaptive design opportunity.

**TABLE 1:** Polymer coating families, useful chemistry, failure vulnerabilities and adaptive design opportunities.

Coating family	Useful chemistry	Main vulnerability	Adaptive design opportunity
Epoxy	Epoxide/amine network; high adhesion and chemical resistance	Residual stress, water uptake, microcracks, cathodic delamination	Barrier reinforcement, inhibitor containers, dynamic interpenetrating networks
Polyurethane	Segmented urethane chemistry; tunable elasticity	Hydrolysis, UV aging, softening during immersion	Disulfide exchange, hydrogen bonding, shape memory, photothermal repair
Acrylic/fluorinated	Weatherability, transparency and surface-energy tuning	Fouling control may conflict with environmental persistence	Fluorine-free amphiphiles, self-renewal, hybrid topcoats
Polysiloxane	Low surface energy, flexibility and fouling release	Weak adhesion to some substrates, mechanical softness	Primer coupling, dynamic siloxane networks, anti-adhesive top layers
Zwitterionic/hydrogel-like	Strong hydration layers and charge balance	Swelling, weak mechanical durability, grafting stability	Dense grafts, interpenetrating networks, tough hydrated top layers
Catechol/polydopamine	Wet adhesion and metal coordination	Oxidation state control, pH sensitivity	Adhesive primers, dynamic coordination, inhibitor-binding interfaces
Bio-derived	Renewable feedstocks and functional groups	Batch variation, hydrolysis, durability trade-offs	Polyphenol chemistry, bio-based epoxies, lignin/tannin additives

## 4 Failure sequence: transport, adhesion, defect propagation and fouling

Water uptake is the earliest and most informative event for many polymer coatings. Water plasticizes polymer networks, changes local dielectric constant, lowers glass-transition temperature, increases segmental mobility and opens diffusion pathways for ions and oxygen. Electrochemical impedance spectroscopy is powerful because coating capacitance can track water uptake while pore resistance and charge-transfer resistance report the transition from barrier degradation to electrochemical activity at the substrate. Work on nanocarbon-rich epoxy composite electrodes is relevant here because it shows how equivalent-circuit interpretation can connect a composite interface to measurable electrical parameters (Cataldo et al., 2020).

Transport coupling follows water sorption. Chloride ions, oxygen, protons, hydroxide ions and organic contaminants do not penetrate independently. Swelling modifies diffusivity; salts change water activity; acids and bases alter hydrolysis and catalyze bond cleavage; local cathodic and anodic regions shift pH; and biological conditioning layers change wetting and nutrient transport. Consequently, a coating that performs well in short NaCl immersion may fail under cyclic UV/salt/humidity exposure or under acidic and alkaline environments. The Bellucci study on graphene epoxy/nanoplatelet thin films exposed to diluted sulfuric acid and KCl is valuable precisely because it treats corrosive media as aging environments rather than as a single endpoint measurement (Bellucci, 2022).

Interfacial weakening is the next stage. Adhesion is not a single property: it is produced by mechanical interlocking, covalent bonding, coordination interactions, acid-base interactions, polar contacts, residual stress and substrate cleanliness. Wet adhesion is generally more relevant than dry adhesion because delamination develops after water and ions reach the buried interface. A chemically elegant coating is weak if it cannot sustain adhesion after water uptake, scratching, pH changes or thermal cycling.

Mechanical defects accelerate all other mechanisms. A scratch, pinhole or abrasive track locally reduces diffusion length to nearly zero and creates a high-flux route for corrosive species. Self-healing chemistry is useful only if it restores the relevant function after such damage. Optical closure is not enough. A scratch that looks closed but does not recover impedance, adhesion or corrosion protection is cosmetic repair. This is why self-healing coatings should report several recovery metrics, including impedance recovery, corrosion current reduction, underfilm corrosion suppression and post-healing wet adhesion.

Fouling amplifies failure in marine, industrial-water and biomedical environments. Organic conditioning films, proteins, polysaccharides, microbial adhesion and biofilm formation modify the coating surface and can create underfilm pH gradients or differential aeration cells. Antifouling chemistry therefore belongs in anticorrosion design, not as a decorative surface property added after barrier optimization. The most credible designs couple an antifouling top layer with an active anticorrosion middle layer and a strong substrate adhesion layer. Graphene oxide-hybridized waterborne epoxy systems provide a representative example in which anticorrosive and antibiofilm functions are deliberately combined within one coating platform (Zhou et al., 2022).

## 5 Barrier reinforcement without a nanocomposite catalogue

Nanofillers can help polymer coatings by creating tortuous diffusion pathways, increasing modulus, improving crack deflection, hosting inhibitors, altering surface energy or providing local conductivity for diagnosis. However, adding more filler is not a design rule. Barrier improvement depends on exfoliation, aspect ratio, alignment, interfacial compatibility, filler loading, cure effects, viscosity and defect formation. A poorly dispersed platelet filler can create microvoids and water channels that reduce protection even when the initial impedance appears high.

Graphene nanoplatelets and graphene oxide illustrate the trade-off. Graphene nanoplatelets can improve tortuosity and mechanical reinforcement, but they can also increase electrical connectivity and galvanic risk if they bridge the coating to the metal substrate. Graphene oxide disperses more easily because oxygenated groups improve compatibility and chemical anchoring, but GO can also increase water uptake if it is not properly modified. Polydopamine-coated graphene oxide provides a representative example of interfacial modification used to reinforce waterborne epoxy coatings for anticorrosion (Cui et al., 2018). Studies on epoxy/graphene nanoplatelet systems are useful because they emphasize real filler morphology, dispersion, contact formation and measurement geometry rather than idealized single-layer graphene (Dabrowska et al., 2014; Plyushch et al., 2016; Ivanov et al., 2017; Bellucci et al., 2018).

Layered double hydroxides, halloysite nanotubes, silica, nanoclays, ZIFs and other containers are often more coating-specific than conductive carbon fillers because they can carry inhibitors or create pH-responsive release. Recent examples include pH-responsive ZIF/GO composite fillers that combine tortuous transport with inhibitor release and measurable self-healing behavior (Wang et al., 2024). The strongest such designs are not those with the most elaborate nanostructure, but those that balance compatibility, release kinetics, water stability, leaching control and reproducibility.

Boron nitride, mica, clay and cellulose nanofibrils offer an important alternative for barrier coatings because they can provide tortuosity without the electrical-conductivity concerns that accompany graphene and carbon nanotubes. This is useful where galvanic coupling is a concern. The design principle is simple: a barrier filler should block transport without introducing a new transport or electrochemical failure pathway.

## 6 Active anticorrosion: inhibitor release, passivation and responsive containers

Passive coatings delay corrosion. Active anticorrosion coatings intervene after corrosive conditions appear. The most common strategy is to store corrosion inhibitors in capsules, layered particles, nanotubes, porous oxides, metal-organic frameworks or polymer domains and release them in response to pH, chloride, redox changes, moisture or mechanical damage. This strategy is attractive because it localizes chemical action where damage occurs, but it also introduces new problems: inhibitor compatibility, premature leaching, capsule rupture during processing, mechanical weakening at high loading and uncertain long-term stability. Reviews of stimuli-responsive self-healing anticorrosion systems and nanotube-based smart coatings likewise emphasize that trigger design, carrier chemistry and repeated protection must be considered together (Fu et al., 2021; Saji, 2022).

The foundational nanocontainer approach used pH-responsive release of inhibitors to provide a self-healing anticorrosion effect in sol-gel coatings (Zheludkevich et al., 2007). Later halloysite and layer-by-layer systems expanded this principle to organic coatings. Recent work has shifted toward smarter containers, greener inhibitors and multifunctional fillers. For example, Alizadegan et al. (2024) developed water-based epoxy coatings containing microcapsules, treated CeO2 particles and 8-hydroxyquinoline derivatives, emphasizing that self-healing and anticorrosion should be evaluated together by microscopy, EIS and salt-spray testing.

pH-responsive fillers are especially logical because corrosion sites generate local pH gradients. A coating may experience alkaline conditions near cathodic oxygen reduction and acidic conditions in confined anodic regions. pH-triggered containers can therefore exploit the corrosion microenvironment itself. However, responsiveness must be quantified. It is not enough to state that a filler is pH-sensitive; release profiles, inhibitor activity, film compatibility, post-release porosity and electrochemical recovery should be measured.

Corrosion inhibitors also create sustainability constraints. Chromates remain historically important but are unacceptable for many modern formulations. Benzotriazole, molybdate, cerium species, 8-hydroxyquinoline, amino acids, plant extracts, tannic acid and other greener inhibitors are frequently investigated. The correct question is not whether an inhibitor is natural or synthetic, but whether it is effective at low dose, retained during service, released locally when needed, compatible with the polymer and safe enough for the target application.

Container chemistry also changes failure risk. A highly loaded carrier may plasticize the matrix, create interfacial voids, release inhibitor too early or weaken wet adhesion. For this reason, corrosion-current reduction should be read together with impedance spectra, cross-section microscopy, leaching data and mechanical adhesion after aging. Nanodiamond, ceria, TiO2, LDH and MOF carriers are valuable only when they improve this complete evidence package rather than merely adding a fashionable filler label (Rahmani et al., 2020; Fadl et al., 2020; Alizadegan et al., 2024; Xu et al., 2024).

The most convincing active systems report release kinetics under relevant pH or ionic conditions rather than only loading capacity. Dual-trigger designs are particularly useful because corrosion sites are not chemically uniform: cathodic areas may become alkaline while anodic regions and hydrolysis reactions can create acidic microenvironments. Dual-pH systems and layered containers therefore offer a rational route to broader response windows (Li et al., 2021; Du et al., 2024). LDH and halloysite containers add ion-exchange or tubular storage, whereas mesoporous silica, MOFs and polydopamine containers provide high loading and chemically tunable pores (Shchukin et al., 2008; Wang et al., 2023; Ren et al., 2023; Xu et al., 2024; Ji et al., 2025).

## 7 Self-healing coatings: reservoirs, dynamic networks and realistic recovery metrics

The modern self-healing-polymer field began with microencapsulated healing agents that released monomer into cracks and restored mechanical function after polymerization (White et al., 2001). Microvascular networks later showed that repeated healing was possible when reservoirs were not exhausted after one event (Toohey et al., 2007). Coatings adapted these ideas by embedding liquid healing agents, drying oils, isocyanates, monomers, catalysts, inhibitors or hydrophobic liquids into polymer films. The advantage is strong local response after damage; the weakness is finite reservoir capacity and possible mechanical degradation when capsules are overused.

Intrinsic self-healing coatings rely on dynamic chemistry inside the polymer network. Diels–Alder adducts, disulfide exchange, imine bonds, boronic ester exchange, hydrogen bonding, host-guest interactions, ionic interactions, metal-ligand coordination and vitrimer-like transesterification have all been explored (Chen et al., 2020). The main advantage is that the healing motif is part of the network rather than a separate additive. The main weakness is that healing may require heat, light, solvent, high humidity, pH change or long times that are not compatible with service conditions.

Recent primary papers show the field moving toward corrosion-responsive and multifunctional healing. Auepattana-Aumrung and Crespy (2023) used responsive polymers with metal coordination bonds to connect self-healing and anticorrosion. Song et al. (2023) used catechol-Fe3+ coordination in an *in-situ* self-crosslinking strategy for autonomous anticorrosion polymers. Yimyai et al. (2023) framed corrosion-responsive self-healing coatings around the stimuli generated by corrosion itself. Sokjorhor et al. (2024) connected dynamic covalent bonds and responsive linkages with self-healing, antibiofouling and anticorrosion behavior. Yang et al. (2025) introduced pH-responsive confined solid-liquid transition of hydrogel microdomains within a coating system to support intrinsic self-healing and anticorrosion recovery.

The practical problem is not lack of chemistry. It is lack of consistent evidence. Many papers demonstrate scratch closure but do not prove barrier recovery. Others show high impedance after healing but without realistic aging before damage. Some test healing under ideal laboratory triggers but not under cyclic salt, UV, humidity and abrasion. A credible self-healing coating should report at least four recovery measures: defect closure, impedance recovery, corrosion inhibition after healing, and retention of adhesion or mechanical integrity.

Intrinsic healing is more elegant but harder to validate. Dynamic covalent bonds and metal coordination can restore continuity without adding a large reservoir, yet their kinetics depend on water, temperature, pH, chain mobility and crosslink density. Catechol-Fe3+ networks, boronic ester exchange, disulfide exchange, imine exchange and supramolecular acid-base interactions should therefore be tested under the same electrolyte and aging conditions used for anticorrosion claims (Auepattana-Aumrung and Crespy, 2023; Boumezgane et al., 2022; Song et al., 2023; Sokjorhor et al., 2024; Yang et al., 2025).

Reservoir-based healing remains important because it can supply fresh material into cracks where intrinsic network mobility is insufficient. Linseed-oil capsules, water-resistant reactive microcapsules and diisocyanate capsules show different philosophies: oxidative polymerization, moisture-reactive repair and polymerization after capsule rupture (Suryanarayana et al., 2008; Sun et al., 2016; Zhang et al., 2024). Their limitation is not the principle of healing but the engineering of capsule size, shell strength, compatibility with the coating, storage stability and retained barrier performance after repeated aging. Table 2 compares representative primary studies by design focus, principal contribution, evidentiary strength and remaining limitation.

**TABLE 2:** Comparative evidence map of selected studies and reviews on adaptive, anticorrosion, antifouling and self-healing coatings.

Evidence block and cited examples	Key finding	Strength	Limitation/next validation step
Reservoir-based healing agents (White et al., 2001; Toohey et al., 2007; Suryanarayana et al., 2008; Sun et al., 2016; Zhang et al., 2024)	Microcapsules or microvascular pathways release monomers, oils or reactive species into defects	Clear local repair mechanism and direct relevance to scratched coatings	Finite reservoir capacity, capsule compatibility, and repeated-aging durability remain limiting
Dynamic and coordination networks (Auepattana-Aumrung and Crespy, 2023; Song et al., 2023; Sokjorhor et al., 2024; Yang et al., 2025)	Reversible metal-ligand, covalent or hydrogel-domain transitions restore continuity without separate reservoirs	Integrates healing chemistry into the polymer network itself	Kinetics depend on water, pH, chain mobility and temperature; service-triggered recovery is still under-tested
pH-responsive inhibitor systems (Zheludkevich et al., 2007; Shchukin et al., 2008; Li et al., 2021; Du et al., 2024; Ji et al., 2025)	Corrosion microenvironments trigger inhibitor release under acidic or alkaline conditions	Mechanistically aligned with local corrosion chemistry	Release profiles, premature leaching and post-release porosity need standardized reporting
MOF/LDH/nanocarrier designs (Rahmani et al., 2020; Ren et al., 2023; Xu et al., 2024; Al Abri et al., 2025)	Porous, layered or particulate carriers combine inhibitor storage with barrier or passivation effects	Adds active protection without relying only on passive tortuosity	Carrier stability, interfacial voids, particle release and manufacturability remain open
Waterborne and lower-VOC epoxy systems (Alizadegan et al., 2024; Ji et al., 2025)	Active corrosion protection is implemented in waterborne epoxy matrices	Improves practical relevance under solvent-reduction constraints	Film formation, water sensitivity and long-term immersion/UV cycling require more data
Antifouling/anticorrosion coupling (Tian et al., 2023; Liu et al., 2024; Li et al., 2024; Sokjorhor et al., 2024; Wang et al., 2025)	Self-healing, fouling resistance and corrosion protection are combined in one coating concept	Moves beyond single-function coating demonstrations	Needs aged-surface fouling assays, abrasion after fouling and standards-aligned corrosion testing
Extreme wetting and liquid-infused surfaces (Tesler et al., 2024; Zheng et al., 2025; Liang et al., 2025)	Surface state, plastron or lubricant layer controls fouling release and water access	Highlights that contact angle alone is insufficient	Plastron collapse, lubricant depletion and roughness damage must be validated
Barrier/EIS metrology and thickness effects (Bierwagen et al., 2003; de Paula et al., 2024; Asperti et al., 2024; Cataldo et al., 2020)	Coating capacitance, resistance and thickness strongly control interpretation of barrier performance	Provides quantitative validation tools for early degradation	Equivalent-circuit models need microscopy, adhesion and exposure-history support
Graphitic-polymer coating precedents (Dabrowska et al., 2014; Plyushch et al., 2016; Ivanov et al., 2017; Bellucci et al., 2018; Bellucci, 2022)	Dispersion, filler contact, measurement geometry and corrosive aging affect reported coating behavior	Useful for processing-property and metrology lessons	Not a substitute for coating-specific multi-stressor validation
Multi-property durability examples (Fadl et al., 2020; Alizadegan et al., 2024; Tian et al., 2023; Liu et al., 2024)	Some studies combine corrosion, mechanical, UV, salt-spray or antifouling elements	Closest current route toward the combined-stressor agenda	Even these rarely integrate all stressors, repeated damage, fouling and leaching in one protocol
Service-specific extensions (Sharma et al., 2025; Menofy et al., 2025; Al Abri et al., 2025)	Marine, cultural-heritage and multilayer metal-protection cases broaden the application space	Shows that coating requirements change with substrate and service environment	Evidence does not always transfer across marine, industrial and mild conservation contexts
Data-driven aging prediction (Chen et al., 2025)	Environmental factors and coating descriptors are used to predict degradation	Useful direction for lifetime assessment and screening	Requires larger standards-aligned datasets with documented exposure histories

### 7.1 Evidence limitations and combined-stressor research agenda

Table 2 intentionally includes studies at different evidence levels. This is a limitation of the field rather than a flaw of any single study: many important papers demonstrate a new healing chemistry, a new inhibitor carrier, a new antifouling surface or a useful diagnostic method under one dominant stressor, but do not yet combine salt, UV, humidity, abrasion, mechanical damage, fouling pressure and release/leaching analysis in one sequence. The strongest current examples move in that direction, including chemically and UV-stable protective coatings, water-based epoxy systems assessed by microscopy, EIS and salt-spray exposure, repeatable self-healing polyurethanes with anticorrosion/antifouling functions, and marine-relevant responsive coatings (Fadl et al., 2020; Alizadegan et al., 2024; Tian et al., 2023; Liu et al., 2024).

The research agenda that follows is therefore constructive. Discovery papers remain valuable, but claims of application-ready adaptive coatings should be staged: first establish chemistry and curing; then quantify baseline barrier and adhesion properties; then expose the coating to standards-based and combined aging; then introduce controlled damage; then measure response by EIS, microscopy, adhesion, corrosion propagation, fouling and leaching. This distinction prevents early-stage chemistries from being dismissed while making clear which data are still needed for translation.

## 8 Antifouling and fouling-release chemistry

Biofouling begins before organisms attach. The surface is first conditioned by water, ions, proteins, polysaccharides and organic matter. Cells and microorganisms then encounter a surface whose chemistry is already altered. This sequence explains why antifouling coatings rely on hydration, low surface energy, surface mobility, steric repulsion, zwitterionic charge balance or controlled release rather than on a single antimicrobial ingredient.

Low-surface-energy fouling-release coatings are commonly based on silicones, fluoropolymers or amphiphilic materials. They do not always prevent initial attachment; instead they reduce adhesion strength so that hydrodynamic shear or cleaning removes organisms more easily. The difficulty is that low-surface-energy coatings may have weak mechanical resistance or adhesion to metals, requiring primers and multilayer architectures. For anticorrosion, the buried primer and mid-layer still determine whether the surface survives if the top layer is abraded.

For superhydrophobic and liquid-infused coatings, the decisive question is stability of the surface state under water. A high static water contact angle in air does not prove durable antifouling or anticorrosion performance. Plastron collapse, lubricant depletion, roughness damage, surfactant contamination and oil loss can all convert an initially impressive surface into a wet, fouling-prone interface. Recent work on plastron thermodynamic stability and durable liquid-infused surfaces therefore provides useful validation criteria for coating papers that rely on extreme wetting states (Tesler et al., 2024; Zheng et al., 2025; Liang et al., 2025).

Zwitterionic coatings based on sulfobetaine, carboxybetaine, phosphorylcholine and related monomers are attractive because they form strongly hydrated layers through electrostatically induced water structuring. Their performance depends on grafting density, chain mobility, charge balance, ionic strength, layer thickness and mechanical anchoring. Hydration-based antifouling avoids biocide release, but it can be mechanically vulnerable and may swell under long exposure. The key is to combine non-fouling surface chemistry with a tough underlayer that protects adhesion and corrosion resistance.

Surface hydration is the central principle behind many non-leaching antifouling coatings. Strongly bound water layers reduce direct contact between proteins, cells or marine organisms and the underlying polymer. However, hydration alone is not enough: the hydrated layer must survive salt, shear, abrasion, UV exposure and contamination by organic conditioning films. This is why antifouling results should be interpreted together with surface chemistry, roughness, swelling and adhesion data, not only with contact angle or short-term bacterial assays (Chen et al., 2010; Lejars et al., 2012; Li et al., 2024; Wang et al., 2025).

Bio-inspired antifouling strategies use catechols, polyphenols, peptides, polysaccharides, lignin, tannic acid, chitosan derivatives or plant extracts. Their appeal is chemical richness and potential lower toxicity. Their risk is variability, uncontrolled release or insufficient durability. Bio-based antifouling should therefore be evaluated by the same criteria as synthetic systems: conditioning-film resistance, bacterial adhesion, algal growth, wet abrasion, leaching, coating adhesion and compatibility with anticorrosion requirements.

The most useful future coatings will not separate antifouling and anticorrosion. In marine and industrial settings, corrosion products can alter surface chemistry and promote biological attachment, while biofilms can create local oxygen and pH gradients that accelerate corrosion. A coupled coating may use a hydrated antifouling top layer, an active inhibitor reservoir in the middle layer and an adhesive primer at the substrate. Graphical abstract illustrates this architecture. Service-specific studies further show that validation requirements vary markedly between marine zinc-polymer protection and self-healing conservation coatings for bronze artifacts (Sharma et al., 2025; Menofy et al., 2025).

## 9 Chemical aging and combined-stressor validation

Accelerated tests are necessary, but they are often misleading when used alone. Continuous salt fog, immersion in a single electrolyte, UV exposure, thermal cycling, acid immersion, alkaline immersion and abrasion each probe only part of the failure sequence. A coating that passes one test may fail when stressors are combined. Bierwagen et al. (2003) showed that EIS is particularly useful during accelerated exposure because it captures coating degradation before obvious macroscopic failure. More recent EIS studies continue to show that coating thickness, filler content and water uptake must be interpreted together (de Paula et al., 2024; Asperti et al., 2024).

International standards provide useful anchors for this validation step. Salt-spray and artificial-atmosphere corrosion tests are commonly framed by ASTM B117 and ISO 9227, while cyclic salt-fog/UV and modified salt-spray protocols can be anchored to ASTM D5894 and ASTM G85. Evaluation after corrosive exposure can draw on ASTM D1654; adhesion can be assessed by ASTM D4541 or ISO 4624; EIS measurements on coated metallic specimens can be aligned with ISO 16773; and paint-system selection and durability concepts can be related to ISO 12944. These standards should be treated as reporting anchors, not as substitutes for coating-specific combined-stressor protocols (ASTM International, 2019; ASTM International, 2021; ASTM International, 2022; ASTM International, 2024; ASTM International, 2026; International Organization for Standardization, 2016; International Organization for Standardization, 2017; International Organization for Standardization, 2018; International Organization for Standardization, 2023).

For adaptive coatings, combined-stressor testing is essential. A self-healing chemistry that works on a fresh scratch may be ineffective after UV aging, plasticization or inhibitor depletion. An antifouling surface that resists bacterial adhesion in a short assay may fail after abrasion or salt aging. A barrier nanofiller that increases initial impedance may create interfacial defects after thermal cycling. Therefore, a chemistry-led validation sequence should include: formulation and curing report, baseline spectroscopy and morphology, dry-film thickness, water uptake or capacitance, initial EIS, aging under combined stressors, controlled damage, response activation, post-healing EIS, adhesion, fouling and leaching.

EIS remains the most transferable diagnostic tool because it is nondestructive and sensitive to water uptake, pore formation, coating resistance, charge-transfer resistance and delaminated area. However, equivalent circuits must be used carefully. A circuit is a model, not proof of mechanism. The selected circuit should be physically plausible, consistently applied and supported by complementary microscopy, adhesion, chemical and mass-transport data. Graphitic and electrochemical studies show the same general lesson: measurement geometry and interface quality can dominate reported behavior, so metrology must be part of material design rather than a postscript (Bellucci et al., 2018; Cataldo et al., 2020).

Salt-spray testing, pull-off adhesion, scribe creep, immersion, contact angle, fouling assays and leaching tests each answer different questions. Salt spray can rank formulations but may not reproduce cyclic service. Contact angle measures wetting but not antifouling alone. Pull-off adhesion measures tensile detachment but may miss underfilm corrosion. A strong paper therefore triangulates metrics and avoids overclaiming from a single test.

Aging prediction is becoming a separate validation problem. Combined UV, humidity, thermal cycling and salt exposure generate nonlinear degradation, so lifetime cannot be inferred by simply extending immersion time. Data-driven and environment-aware degradation models are useful when they are grounded in physically meaningful descriptors: temperature, humidity, UV dose, electrolyte composition, coating thickness, substrate preparation, impedance parameters, adhesion loss and scribe creep. Such models should complement, not replace, mechanistic coating tests (Chen et al., 2025).

## 10 Sustainability, safety and regulatory direction

Protective coatings are sustainability tools because they extend service life, reduce maintenance and prevent corrosion-related material losses. They can also create sustainability problems if they use persistent biocides, hazardous inhibitors, high-solvent formulations, difficult-to-recycle multilayers or poorly contained nanomaterials. A modern adaptive coating must be judged by net service benefit, not only by laboratory performance.

Waterborne and high-solids formulations reduce volatile organic compounds, but they introduce formulation challenges: film formation, coalescence, water sensitivity, dispersion stability and cure compatibility. Bio-based resins and additives reduce fossil feedstock dependence, but their durability must be proven. Fluorine-free fouling-release systems are desirable, but they must match the performance of incumbent low-surface-energy coatings. Green inhibitors are attractive, but they must be released at the right time and remain compatible with the network.

Nanomaterial safety also matters. Graphene-based materials, CNTs, nanoclays, silica, layered hydroxides and metal oxides may be safe when immobilized in a stable coating but problematic if released during abrasion, weathering or disposal. The safety question is therefore exposure-specific. A responsible manuscript should report whether the additive is bound, whether leaching or particle release is expected, how abrasion debris is handled, and whether the coating contains substances of regulatory concern. The safety assessment of graphene-based materials has emphasized that material form, functionalization, dose, exposure route and persistence must be considered together (Fadeel et al., 2018).

Safe-and-sustainable-by-design thinking should enter at formulation stage. The hierarchy is straightforward: avoid hazardous ingredients where possible; immobilize functional additives; minimize release while enabling local response; validate performance under realistic aging; assess end-of-life or repair routes; and compare with incumbent coatings. A coating that performs slightly better in a laboratory test but introduces a poorly controlled release pathway may not be a genuine improvement.

## 11 Design rules and reporting recommendations

The following design rules summarize the review. First, define the dominant service environment before selecting chemistry. Marine immersion, atmospheric corrosion, acidic industrial exposure, biomedical fouling and humid electronics packaging require different balances of barrier, adhesion, self-healing and surface hydration. Second, distinguish barrier improvement from adaptive response. A filler that raises initial impedance is not equivalent to a responsive inhibitor container. Third, separate optical crack closure from electrochemical recovery. Fourth, treat wet adhesion and underfilm corrosion as central variables. Fifth, test antifouling after surface aging, abrasion or salt exposure, not only on fresh surfaces. Sixth, report dry-film thickness, roughness, cure history, substrate preparation, scratch geometry, electrolyte composition and replicate statistics.

A balanced review of adaptive coatings should use graphitic-polymer and nanocomposite studies only where they illuminate coating-specific questions: dispersion, processing, electrical or electrochemical characterization, thermal transport, corrosive aging and measurement artefacts. The main weight should remain on anticorrosion, antifouling, self-healing, wet adhesion, aging and validation studies. That balance keeps the manuscript centered on polymer and surface chemistry rather than on a catalogue of carbon-based multifunctional materials.

Figure 2 can be used as the practical checklist for future papers. A strong coating study should move from chemistry to evidence: synthesis and curing, structure and morphology, baseline impedance and adhesion, combined aging, controlled damage, response measurement, post-healing corrosion protection, fouling resistance and safety. Papers that report only a high initial impedance modulus, a large contact angle or a visually healed scratch should be treated as early-stage demonstrations. Table 3 translates these design rules into failure modes, preferred chemistry and minimum validation requirements.

**FIGURE 2:**
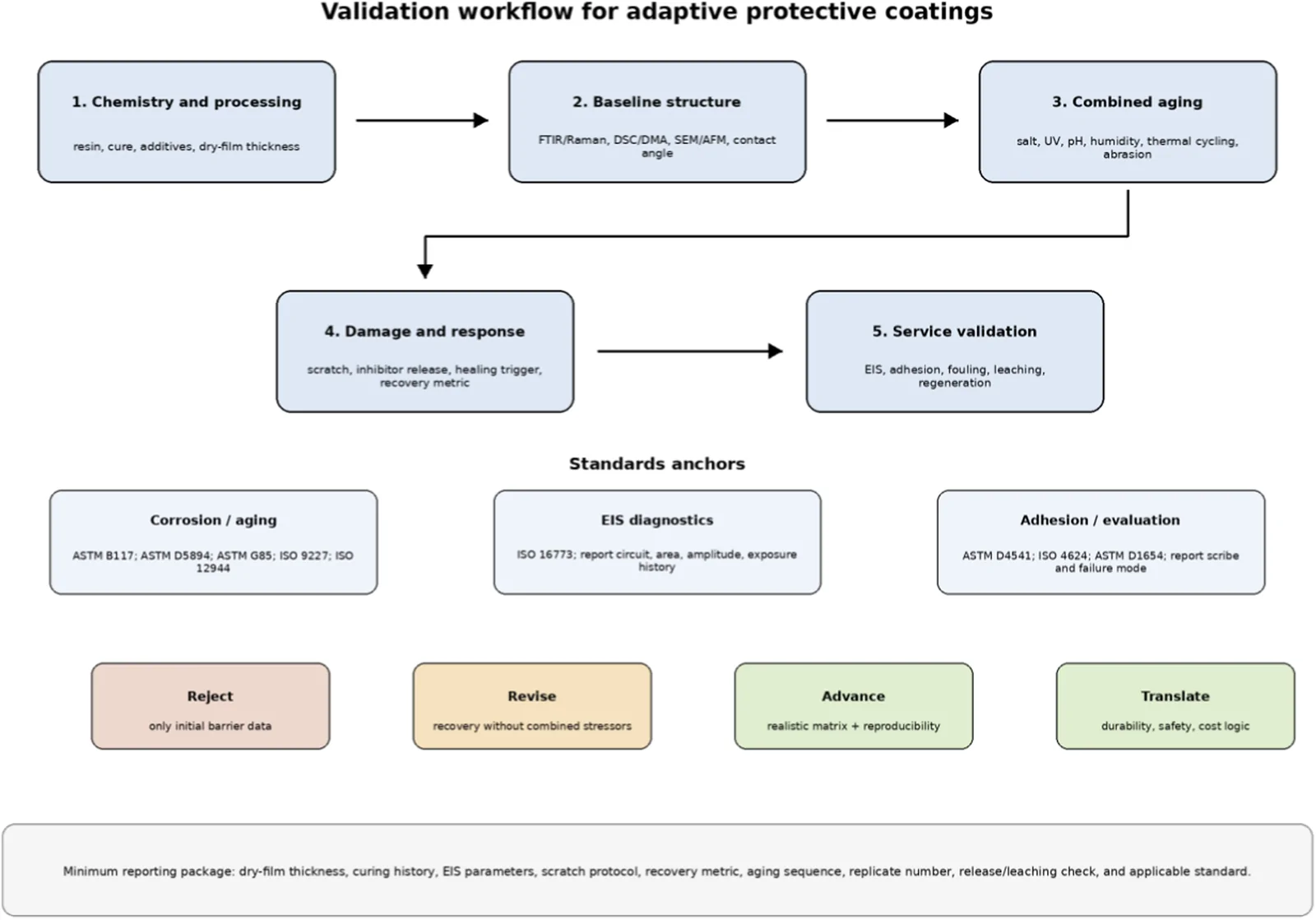
Validation workflow for adaptive protective coatings. The workflow separates discovery-level coating chemistry from application-relevant evidence, includes representative ASTM/ISO validation anchors, and highlights the minimum reporting package needed for review-level benchmarking.

**TABLE 3:** Failure modes, mechanistic origins, preferred chemical strategies and standards-aware minimum validation requirements for adaptive protective coatings.

Failure mode	Mechanistic origin	Preferred chemistry	Standards-aware minimum validation
Barrier failure	Water, oxygen and ion permeation through pores/free volume	Crosslink density control, tortuous fillers, lower water uptake	EIS capacitance/resistance (ISO 16773), immersion, permeability, salt spray (ASTM B117/ISO 9227)
Wet adhesion loss	Hydrolysis, interfacial water, residual stress, cathodic delamination	Primer chemistry, catechol/coordination adhesion, substrate pretreatment	Pull-off after aging (ASTM D4541/ISO 4624), scribe creep, wet adhesion
Local corrosion after scratch	Direct electrolyte access and pH gradients	pH/redox/chloride-responsive inhibitor release	Scribed EIS, salt immersion, local microscopy, coating evaluation after exposure (ASTM D1654)
Crack propagation	Mechanical damage and thermal cycling	Dynamic covalent/supramolecular healing, capsules, vascular networks	Optical closure plus impedance and corrosion recovery after controlled damage and cyclic aging
Biofouling	Conditioning films and microbial adhesion	Zwitterionic/hydrogel-like hydration, fouling release, non-leaching surfaces	Protein/bacterial/algal assays, abrasion after fouling, aged-surface tests
Secondary contamination	Inhibitor, biocide or nanomaterial release	Immobilization, greener inhibitors, leaching controls	Release profiles, abrasion debris, leaching controls, end-of-life logic

## 12 Conclusion

Adaptive polymer-based protective coatings are best understood as chemical interfaces that regulate a sequence of coupled events: water uptake, ion transport, interfacial weakening, mechanical damage and fouling. Their value lies not in the presence of a fashionable additive or a high initial performance metric, but in the ability to preserve or recover function under combined stressors. This shifts coating design from passive barrier formulation toward molecular and interfacial engineering of transport, adhesion, release, healing and surface hydration.

The strongest current directions include pH-responsive inhibitor containers, dynamic covalent and metal-coordination networks, waterborne epoxy self-healing systems, zwitterionic and hydrogel-like antifouling layers, bio-derived additives, durable liquid-infused surfaces, and multilayer architectures that separate adhesion, barrier, active response and surface non-fouling. Earlier graphitic-polymer work contributes useful precedents for processing, dispersion, electrochemical and electromagnetic characterization, thermal transport and corrosive aging, but the broader field must be assessed through coating-specific evidence.

The next stage should be more demanding. Coating papers should report dry-film thickness, cure history, filler dispersion, aging sequence, scratch protocol, impedance recovery, wet adhesion, fouling resistance, inhibitor release or leaching and safety considerations. Only then can adaptive coatings move from attractive laboratory concepts to reliable protective systems for marine, industrial, biomedical, infrastructure and electronic applications.
